# The prevention of – and first response to – injuries in Nepal: a review of policies and legislation

**DOI:** 10.1186/s12961-021-00686-1

**Published:** 2021-04-14

**Authors:** Puspa Raj Pant, Julie Mytton, Milan Raj Dharel, Amrit Dangi, Writu Bhatta Rai, Sunil Kumar Joshi

**Affiliations:** 1 Faculty of Health and Applied Sciences, University of the West of England Bristol, Bristol, BS8 1NU UK; 2 Bristol Medical School, Centre for Academic Child Health, University of Bristol, Bristol, BS8 1NU UK; 3 Faculty of Health and Applied Sciences, University of the West of England Bristol, Bristol, BS16 1QY UK; 4 Swatantrata Abhiyan Nepal, Bakhundole, Lalitpur, Nepal; 5 Department of Community Medicine, Kathmandu Medical College, Kathmandu, Nepal

**Keywords:** Injury prevention, First response, National policies, Legislation, Law implementation, Nepal

## Abstract

**Background:**

Injuries, the cause of an estimated 4.5 million deaths annually and many more disabilities worldwide each year, are the predictable outcome of particular circumstances. One of the most effective ways to prevent injuries is through policy and legislation. The aim of this research study was to identify and critically review all policy and legislation in Nepal that had the potential to prevent injuries.

**Methods:**

We identified legislation and policy that met inclusion criteria through a stakeholder meeting, networks and contacts, and websites and electronic resources. Each included document was critically reviewed to identify areas of strength and opportunities for improvement. We compared the included documents against WHO’s recommendations of known effective interventions.

**Results:**

Sixty-two documents met the inclusion criteria for this review. Of these, 24 (38.7%) were exclusively related to road injuries, 11 (17.7%) to occupational injuries, 6 (9.7%) to injuries in the home and 5 (8.1%) to injuries at school; 30 (48.4%) documents included text related to the first response to injuries. Of 127 strategic recommendations by WHO that provided an area for policy or legislative focus, 21 (16.5%) were considered adequately met by Nepali policy and legislation, 43 (33.9%) were considered partially met and 63 (49.6%) were not met.

**Conclusion:**

We drew five conclusions from this critical policy review, which we have related to recommendations as follows: widening the scope of legislation and policy for injury prevention to emphasize injuries occurring at home or school; addressing the causes of injuries and promoting proven preventive measures; greater clarity on both individual and institutional roles and responsibilities; trustworthy data and quality evidence to inform decision-making; and financial investment and capacity-strengthening for injury prevention and first response. The current system of federal governance in Nepal has potential for strengthening injury prevention and first response at the central, provincial and local levels.

**Supplementary information:**

is available for this paper at 10.1186/s12961-021-00686-1.

## Background

Injury is defined as “*unintentional or intentional damage to the body resulting from acute exposure to thermal, mechanical, electrical, or chemical energy or from the absence of such essentials as heat or oxygen*” [1]. Injuries are usually the predictable outcome of particular circumstances, and are therefore largely preventable. In the year 2017, deaths due to all injuries combined were estimated to be 4.5 million worldwide [2]. Historically, over 90% of the total global injury burden lies in low- and middle-income countries [3]. The Global Burden of Disease (GBD) study estimated that 16 831 people (95% uncertainty interval 13 323–20 579) died from injuries in Nepal in 2017. The proportion of all deaths due to injuries increased from 6.31% in 1990 to 9.2% in 2017 [4]. According to GBD estimates for Nepal, disability-adjusted life-years attributed to injuries increased from 881 452 (668 625–1 049 545) in 1990 to 897 969 (718 350–1 092 186) in 2017, including 715 602 (540 423–894 816) years of life lost due to injury [4]. Population-based estimates of injury incidence are available but use variable definitions and data sources. A systematic review in 2019 of injury research in Nepal identified a large number of publications, though most were small hospital case series [5] at high risk of bias. The most recent nationally representative community-based survey used data from the 2001 census and reported an annual incidence of fatal injuries of 42/100 000 in men and 19/100 000 in women [6]. In addition to physical or emotional outcomes, injuries may result in financial consequences. A survey in 2019 found that 60% of all injured persons incurred direct costs associated with injuries of up to 350 000 rupees (US$ 3181.82) [7]. It is also evident that injury significantly increased the risk of catastrophic expenditure, irrespective of Nepali household economic status [8]. The rate ratio for injury-related expenditures for the households in the poorest quintile was 1.19 (95% CI: 0.35–4.03), with significantly increased risk of catastrophic expenditure (second highest in quintiles 2–5) by wealthier households [10].

The economic burden of injuries is huge, especially for low- and middle-income countries [9, 10]. While the economic burden of all injuries in Nepal is not known, the cost of road injuries alone was estimated at US$ 122.88 million in 2017, which was 1.52% of the gross domestic product, or US$ 7.56 billion at constant gross national income for that year [11].

Risk factors for injuries vary according to the type of injury. Possible reasons for the increased incidence of and mortality due to transport injuries in Nepal may be related to challenging road conditions, with a vast expansion of road networks and rapidly increasing volume of vehicles, and inadequate investment in road maintenance, road safety and law enforcement arrangements [4]. WHO has stated that occupation, alcohol and substance use, socioeconomic factors, medications, underlying medical conditions such as loss of balance and poor vision, and environmental factors are all associated with the risk of fall injuries [12]; Nepalese females have greater exposure to injury risks associated with household chores, and in particular from cooking and collecting firewood and animal fodder, which can involve climbing trees [13]. People living in poverty are at increased risk of all types of injuries [14]. Natural hazards also cause injuries in Nepal; the massive earthquake and its aftershocks in 2015 killed approximately 9000 and injured over 22 000 people [15, 16], and people die in landslides and flooding during the monsoon season every year. Different types of injuries have different risk factors, mechanisms and consequences, and therefore multisectoral and multidimensional approaches are needed to achieve effective injury prevention. Improving access to high-quality first response care to victims of injury, followed by good medical treatment, has the potential to save lives, reduce injury severity and prevent disability [17]. The World Health Assembly has urged WHO Member States to comprehensively assess their prehospital care capability and include it in emergency response plans [18]. Nepal’s Health Sector Strategy Implementation Plan 2016–2021 [19] included an expansion of first response services (or public health emergency preparedness), and the National Health Policy is formulated addressing this agenda [20].

Over the last three decades, Nepal has experienced continuous political transitions, from an absolute monarchy (present until 1990), through a decade of armed conflict from 1996 to 2006, to establishment of a federal republic since 2015. As specified in the Constitution of Nepal 2015, the country now has a three-layer federalized system of governance, at the federal (central), provincial and local levels [21]. Despite political changes, over the period since 1990, Nepal has recorded a steady decline in mortality, resulting in considerable improvement in the health of the population [22].

Recognizing injury as a global public health concern, WHO developed guidance for decision-makers involved in developing policies on violence and injury prevention [23] and for health ministries, emphasizing the public health approach to violence and injury prevention [24]. A strong political commitment and coordinated efforts are equally important [25, 26] along with educational, environmental and health policy development [27]. Multisectoral injury prevention policies are best addressed alongside relevant health care policies [28]. One definition of injury prevention policy is “*a rule or decision having the capacity to guide or determine the actions of individuals, groups, organizations or governments with the goal of affecting the surveillance, risk, incidence, severity, disability, cost or other aspects of injury*” [29].

Enforcement of policies and legislation has been proven to reduce the incidence of injuries through behaviour change and the creation of safer environments that complement education and environmental change interventions [30]. However, a sound plan is needed for their implementation and enforcement. Globally, there is general agreement among public health practitioners, policy-makers and human rights activists that there is a need for improvements in policies and adequate investments both for injury prevention and to establish stronger health systems to provide care for patients with injuries [31–34].

Research can identify “what works” to prevent injuries and “how to” implement effective prevention interventions at a national level. Such evidence can guide those who develop policies, laws and regulations [23, 30]. To formulate effective injury prevention policy, a critical review of existing policies and legislation that support injury prevention and control is important. This study aimed to provide an overview of existing policies and legislation in Nepal that support the prevention of – and the effective first response to – injuries. This would enable the identification of areas of strength and opportunities for policy development, identify priority areas for advocacy, and support federal, provincial and local governments and policy-makers in developing informed and effective policies that prevent injuries and reduce risk factors for injuries.

## Materials and methods

The theoretical framework for this review was the Centers for Disease Control and Prevention’s policy analytical framework [35, 36]. Whilst this framework is typically used to evaluate an individual policy, we adapted the first three stages of the framework for the purpose of collating, synthesizing and critiquing a body of policy on one topic. Stage 1 (problem identification) established the rationale for the review: the lack of clarity regarding the existence of effective legislation and policy in Nepal to prevent injuries and enable effective first response to injuries. Stages 2 (policy analysis) and 3 (evaluation of policy development) required the identification, analysis and synthesis of any current legislative or policy document meeting prespecified inclusion criteria. To be included in the study, the laws and policies needed to have the potential to influence risk factors for unintentional injuries or influence the first response to such injuries. We specifically sought laws and policies that had the potential to influence the occurrence of injuries in four settings where the majority of unintentional injury occurs: on the road, at home, in the workplace and at school. The term “legislation” is defined in this review as “*a law which has been promulgated by a legislature or other governing body or the process of making it*”. We defined “policy” as “*a deliberate system of principles to guide decisions and achieve rational outcomes. A policy is a statement of intent, and is implemented as a procedure or protocol.*” Hereafter, in this paper, we use the phrase “policy document” to denote both laws and policies.

To identify relevant policy documents, we applied a scoping review methodology described by Arskey and O’Malley [37]. This approach enables (i) the identification of all legislative and policy publications meeting the inclusion criteria through an iterative process, (ii) summarizing of the content of included policies and (iii) identification of policy gaps where there are opportunities for policy development. We identified potentially eligible documents from a broad range of sources. A stakeholder consultation event involving representatives from key national agencies identified sources of documents for inclusion and the individual or institutional owner and/or gatekeepers of such documents. This event enabled the development of a list of ministries, departments and commissions, which were followed up through contacts and website searches. Potentially eligible documents were screened by two researchers (AD and PRP). Where it was unclear whether the document met the inclusion criteria, documents were discussed with the wider research team. We excluded (i) documents describing only individual views or expert opinion, (ii) documents that only addressed intentional injuries (suicide, assault, homicide) or injuries arising from natural disasters, (iii) policies and legislation that have been superseded or fall outside the jurisdiction of the Government of Nepal and (iv) documents reporting only the volume of injuries, risk factors or cost burden of injuries, or the evaluation of injury prevention interventions, or the impact of policies.

We developed a data extraction template informed by the framework described for the assessment of child injury prevention policies (A-CHIPP) by Alonge et al. [38]. For each included document, we extracted information for the following fields: the type of policy document, the source agency, the enforcing authority, the date of production and the status of implementation. Information on injury prevention and first response were extracted under the following five headings: (1) scope of injury types included, (2) risk factors addressed, (3) prevention measures, (4) first response measures and (5) institutional arrangements (for example, factors supporting implementation, including but not limited to leadership, data systems, technology, workforce). We also collected data on the location of the injury event being addressed (on the road, at home, work or school) and whether the document addressed the first response to injuries. Data were collated separately for legislation and policies. This enabled both quantitative (such as number of documents by injury type) and qualitative (such as assessment of scope, use of evidence) analysis of the included policy documents in a manner recently described as policy content analysis by Hall and Steiner [39]. To compare the included injury prevention and first response policies and legislation to international best practice, we considered the degree to which the included documents supported, partially supported or failed to support WHO’s recommendations of known effective interventions to prevent injuries. Evaluation of the effectiveness of individual legislation and policy documents was outside the scope of this study.

**Fig. 1:**
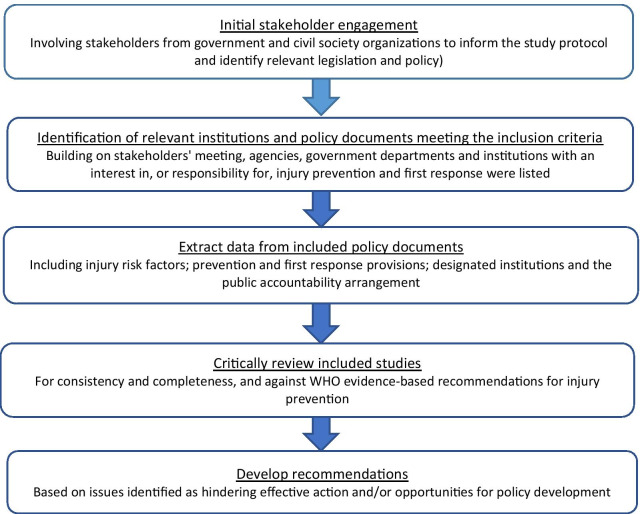
Flow of policy document review activities

A chronological summary of all included studies was constructed as described by Conway et al. [40]. The findings are presented categorized by the location of the injury event (by road, home, workplace or school), because national prevention strategies and implementing authorities tend to focus on location, and this format was considered the most useful for decision-makers. Recommendations were formulated based on areas where there was a gap between internationally recognized and proven practice, and the existing national policy documents. The purpose of the recommendations is to encourage discussion leading to strengthening of policies in those areas where current legislation is limited or contradicted, or where institutional arrangements are asymmetrical. A summary of the methods used in this study is provided in Fig. 1, adapted from the six steps for evaluation described by the Centers for Disease Control and Prevention [36].

## Results

**Fig. 2:**
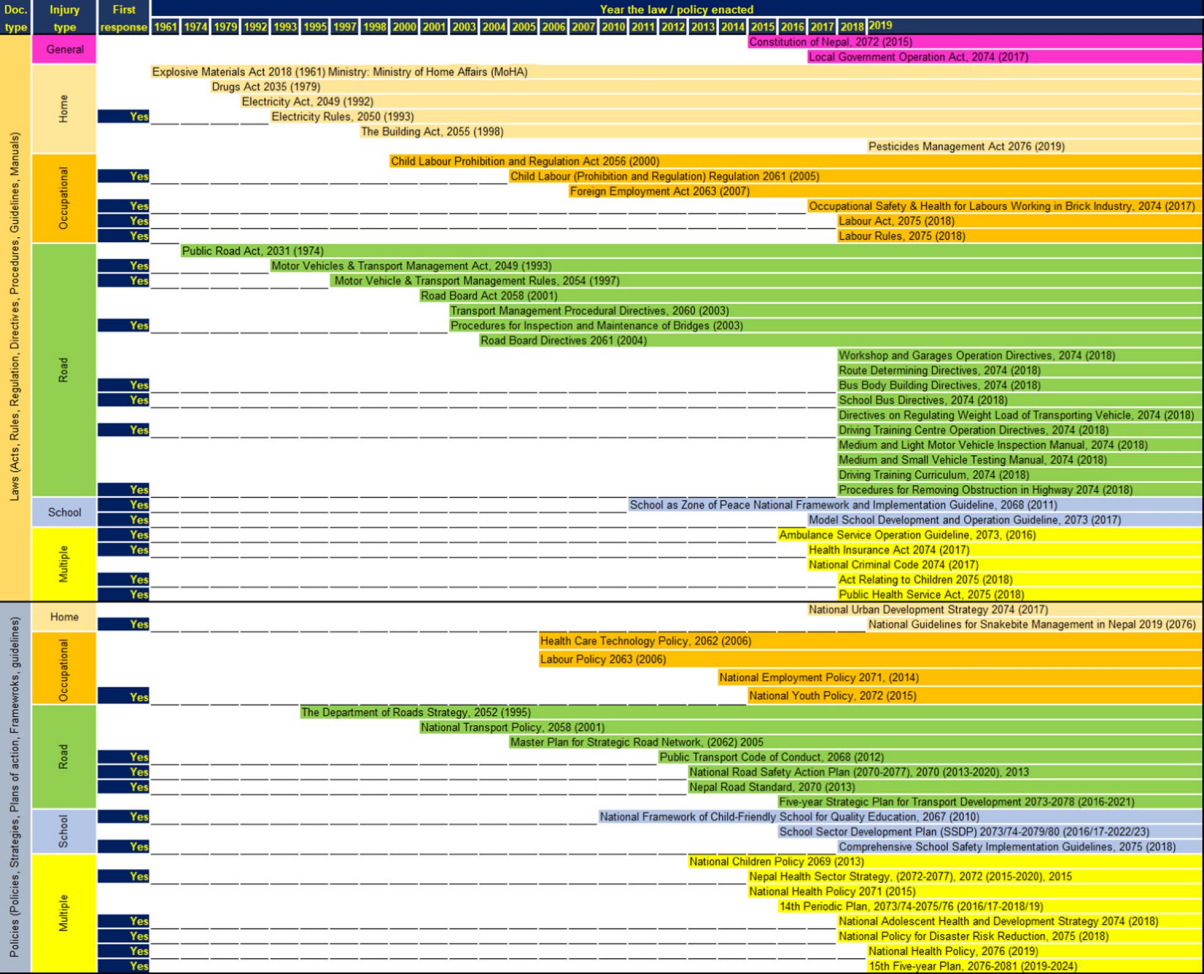
Timeline and visualization of included documents by types and injuries. The category “Multiple” is for the documents that cover more than one type of injury

We identified 127 potentially eligible documents, of which 62 met our inclusion criteria (Additional file 1). Included documents varied markedly in the level of detail describing legal/policy provisions for injury prevention and first response. Figure 2 visualizes the included documents by years, types and injuries.

**Fig. 3:**
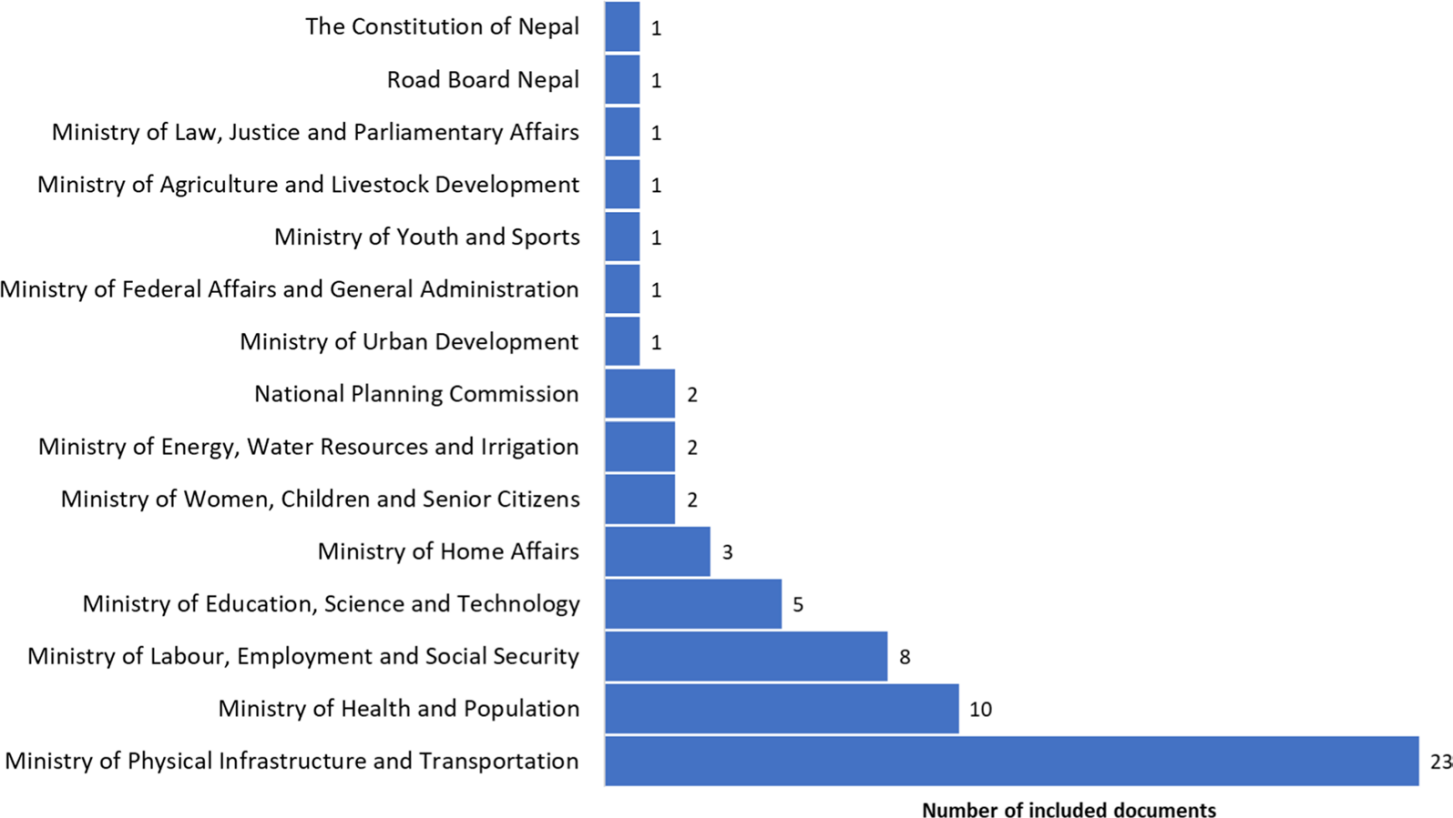
The lead ministries for the creation of laws and policies included in this review

The review categorized documents according to their scope. Among 62 included documents, those relating exclusively to one injury type included 24 (38.7%) related to road injuries, 11 (17.7%) to occupational injuries, 6 (9.7%) to injuries in the home and 4 (6.5%) to injuries at school. Sixteen documents included text related to injuries occurring in more than one location or to injuries in general, and 30/62 (48.4%) documents included text related to the first response to injuries. The laws and policies included in this study originated from 15 agencies including 12 ministries (Fig. 3).

### Constitutional provisions

The Constitution of Nepal and the Local Government Operation Act, (2017) provide overarching legal guidance at the federal, provincial and local government levels. Seven articles (Article 16, 30, 35, 39, 42, 44 and 51) of the Constitution of Nepal either explicitly state or have implications that impact on issues related to injuries, trauma and physical harms from external agents (exact excerpts in the constitution are compiled as Additional file 2). In order to conform to the constitutional provisions stipulated by these articles, the state formulated, revised and/or replaced legislative and policy instruments. According to the provisions of the Constitution of Nepal, the provincial and local governments have delegated authority and responsibility to both make laws and implement activities through the Local Government Operation Act (2017). These also include activities related to the prevention and control of injuries (exact excerpts in the Act are compiled as Additional file 2).

### Laws related to road injuries

We identified 21 legislative documents related to road and transport management. The Motor Vehicles and Transport Management (MTVM) Act 1993 is the main document supporting road traffic injury prevention and control. The preamble of the Act reads: “*… it is expedient to make transportation services consolidated, efficient and effective with a view to preventing motor vehicle accidents, enabling the victims of accidents to have compensation, providing for insurance and making transportation facilities available to the public generally in a simple and easily accessible manner*.” Chapter 7 of the Act describes most of the traffic rules including one related to the use of seat belts and motorcycle helmets: “*(1) While driving a motor vehicle as prescribed, the driver and a person seated on the front seat of the motor vehicle shall use the seatbelt. (2) While driving a motorcycle or similar other two wheeled motor vehicle, the driver and the pillion rider shall use helmets.*” (Section 130, Articles 1 and 2).

Most other documents have been originated through the provisions of this Act. Other key documents include the MTVM Rules 1997 and the Transport Management Operational Directives 2003. These two instruments are further supported by several bylaws: Driving School Curriculum 2012, Driving Testing Centre Operation Directives 2018, Bus Body-building Directives 2017 and School Bus Directives 2017. These instruments have further facilitated the formulation of policies.

### Policies related to road injuries

We identified 14 policy documents that included road traffic injuries or their prevention. Most policy documents focused on speed control, road surface construction standards, the conduct of road safety audits, the formation of a road safety council, effective transport monitoring and surveillance, and the dissemination of key road safety messages. Overall, we noted little mention of implementation or enforcement of these provisions.

The National Transport Policy 2001 has the stated objective of making the transport system sustainable, reliable, safe, well-facilitated and independent. In Action Plan 6.4 of this policy, it is stated that “*[this policy] recommends establishing a Road Transport Authority as well as a National Transport Board envisaging increased coordination among all authorities related to Transportation and transport management.*” The Public Transport Code of Conduct 2011 includes rules to be followed by drivers, conductors, vehicle owners and passengers. The Nepal Road Safety Action Plan 2013–2020 outlined action plans across WHO’s five pillars of road safety [41], including possible preventive methods, suggested institutional mechanisms (e.g. the establishment of a road safety council) and considered workforce capacity issues. However, none of the plan has been implemented. Road safety was prioritized as a major programme in the 14th Periodic Plan 2016–2018 and the 15th Five-Year Plan 2019–2024, where the Government of Nepal specified “*the requirement of road safety audits at all phases of design, construction and maintenance of roads*” (Strategic Plan: Key Programme A10 and A11). The strategic plan also articulated some time-bound targets for “transport management areas” in Section E, which include the use of intelligent traffic systems to manage traffic, and the implementation of a road accident information management system (RAIMS). The Ministry of Health and Population (MoHP)’s National Health Policy 2019 identified road traffic accidents as a major problem for health services in Nepal. The National Policy for Disaster Risk Reduction 2018 recommended the establishment of a road safety council. Such a council has yet to be established.

The current 20-year Master Plan for Strategic Road Networks (2002–2022) mentions “*road crash reduction to achieve the vision of national integration and socioeconomic development*” (Chapter 2, Outcome 7). However, we found that most of the included policy documents failed to mention monitoring and evaluation of performance to assess whether objectives have been met, nor did they specify what corrective action should take place if objectives were not achieved. Similarly, many documents lacked inclusion of issues relating to administration, named responsible institutions, time frames or allocated resources (budget, human resources, equipment), limiting their ability to be implemented.

### Laws related to injuries at home

Altogether 12 legislative documents were related to home injuries and home safety, produced by seven ministries. These documents included electrical safety (Electricity Act 1992, Electricity Rules 1993), fire safety (Explosive Materials Act 1961), drug safety (Drugs Act 1978) and pesticides (Pesticides Management Act 2018), as well as children’s safety at home (Act Relating to Children 2018). Accordingly, the Electricity Rules have provisions for “Safety measures regarding electric devices/appliances” in Chapter 5, particularly Sections 52, 58, 60–62 and 67. For example, Section 61 specifies the safety of internal wiring: “*Wiring method and the electric devices installed for the safety of internal wiring of the consumer’s house shall have to be in accordance with the prevailing technical standard*.” The legislation relating to electrical and fire safety primarily considers public premises. The Explosive Materials Act 1961 is practiced largely to control fireworks activities during festivals on the basis of its Sections 4 and 5, which prohibit the production, sale and transport of explosive materials. The Building Act 1998 originated from the concept of right to safe housing as mentioned in the constitution and International Covenant on Economic, Social and Cultural Rights (ICESCR). The preamble of the Building Act reads, “*…whereas, it is expedient to make necessary provisions for the regulation of building construction works in order to protect building against earthquake, fire and other natural calamities, to the extent possible*.”

### Policies related to injuries at home

A total of six policy documents addressed home injuries in some way, among which only two were related exclusively to this location. The documents lack specific plans but mostly recommended following the Nepal National Building Codes to make homes and shelters safe. These documents are the 14th and 15th National Plans, National Policy for Disaster Risk Reduction, National Children’s Policy and National Urban Development Strategy. However, the National Building Codes take the form of provisional recommendations only, which do not legally obligate citizens to follow these policy provisions. The Department of Urban Development and Building Construction (DUDBC) is the main authority tasked with formulating policy documents for preventing home injuries. Other organizations developing policies in this area include the National Planning Commission, the MoHP and the Ministry of Home Affairs. The National Children’s Policy 2013 includes the statement that homes should be made “child-friendly” but does not state how this should be achieved. The National Guidelines for Snakebite Management (2019) include a separate chapter on the prevention of snakebites; it lists very useful advice on keeping houses safe by eliminating places where snakes can live. Most of the policy documents on home injury did not include detailed information on prevention of injuries, vulnerable groups, implementation of the law/policy, governance or financing of the programme.

### Laws related to injuries at school

We found only five legislative documents related exclusively to school injuries. These included the Local Government Operation Act 2017, Model School Development and Operation Guideline 2017, the School as Zone of Peace National Framework and Implementation Guideline 2011, the Act Relating to Children 2018, and the Building Act 1998. The Model School Guideline (2017) specifies the criteria to be met for a school to be called a model school: “*a school must have safe structure and must be located in safe places with proper fencing or compound*” (Criteria 4.2) and “*a school must have first aid kit, trained first aid care providers and referral system*” (Criteria 5.2). The Act Relating to Children 2018 requires the governments at the federal, provincial and local levels to adopt measures for the protection, safety and health of children, with a specific focus on children in need of special protection. Children with disabilities and others considered to be at increased risk are recognized and provided special protection. However, the rules and directives needed for effective enforcement of the laws and provisions listed in the Act are still to be formulated.

### Policies related to injuries at schools

Nine documents reported school safety. The majority of these were formulated after 2015, which suggests that the need for improved school safety may have been highlighted by the earthquakes that year. This study found that most of the documents identified earthquakes (or disasters) as a major risk factor for injuries at school. However, many did not consider other potential hazards, for example, national standards and requirements for playgrounds (size, surfacing, equipment, player limits, play stacks), furniture (height/width, shape, quality, quantity), classrooms (size, capacity, ventilation, air condition), school building (school site requirements, number of classrooms in schools, emergency exits) or electrical requirements (power supply, lighting, electrical panels).

The National Health Policy recommends the availability of at least one health professional in each school, which may be helpful for preventing injuries at school (Section 6.5.2). The School Sector Development Plan (SSDP) seeks to ensure safe learning environments and to mainstream comprehensive school safety and disaster risk reduction in the education sector. This is through the strengthening of school-level disaster management and resilience planning (SSDP Sections 3.2 and 7.4). School improvement plans should identify a minimum package of school safety and promote the development and monitoring of a standard set of key messages. Subsequently, the Comprehensive School Safety Minimum Package 2018 was formulated, which facilitates the implementation of the SSDP (2016–2023). In the introduction section of the package, it is recommended that “*schools implement other measures to aim for maximum school safety to (i) protect students and teachers from death, injury and harm in school*” (Section a.3). The document envisages “*improving safety of schools through physical infrastructure construction and retrofitting*” (Section b.1). The national framework of child-friendly schools for quality education was formulated in 2010 and provides minimum and expected indicators for a child-friendly school, a school environment in which children are motivated and able to learn because it is made safe, and teachers are friendly and welcoming [42]. This document stated that “*as it is imperative to pay attention to the health, safety and protection of children within school premises, a child-friendly school should collaborate with families and the community for the health, safety and protection of children. It is because the health of children also depends on the environment outside the school*” (Framework Section 2.5). Despite these documents promoting safety in school settings, there is a lack of implementation plans on how safety should be ensured and who is responsible for actions.

### Laws related to occupational injuries

We found nine legislative documents related to injuries that occur in the workplace. The Labour Act 2018 and Labour Rules 2018 contain several provisions on occupational safety, including explaining the laws, standards and safeguarding measures explicitly for preventing injuries in the workplace. There is a bylaw to offer protection for workers in brick factories called the Directives for Occupational Safety and Health for Labourers Working in the Brick Industry 2017.

The Labour Act 2018 has defined provisions for all categories of workers, and mandates that organizations with more than 20 employees formulate an occupational safety and health policy for their workplace. In this Act, the duties of employers towards their workers and nonworkers are provisioned in Sections 69 and 70, respectively. Some points in these sections are exclusive for workplace injuries: “*Create safe environment through the management of workplace safety and health*” (Section 69.1a); “*make arrangements ensuring no adverse effect on workers from use, operation, storage or transport of chemical, physical or biological liquids*” (Section 69.1b) and “*employer shall make necessary arrangements ensuring no adverse effect to those who come and go to workplace or those who pass by workplace*” (Section 7.1). Specific risks to the safety and health of female workers in the event of pregnancy including the care of children of workers is included within the Labour Act (Section 81.2) and Labour Rules (Chapter 4, Rule 17). However, neither of these documents defines penalties or punishments for violation of occupational safety provisions, or considers the specific risks to the safety and health of workers with disabilities.

The review identified that in addition to the Ministry of Labour, other ministries supporting occupational safety through legislation included the MoHP and the Ministry of Energy, Water Resources and Irrigation (MoEWRI). Whilst the legislation produced by the Ministry of Labour is generic for all workers, legislation from the MoHP and MoEWRI highlighted safety issues for specific workers. For example, the Public Health Service Act 2018 explains occupational safety laws for health professionals, whilst the Electricity Rules 1993 highlight occupational safety laws for electrical workers. Designated responsibilities are clear: the labour office has the primary responsibility of enforcing the provisions, whereas the employers have the responsibility of ensuring the safety of their employees.

### Policies related to occupational injuries

We found 11 documents that reflected policy provisions regarding occupational safety and health, though none of these documents explicitly explained occupational safety policies or plans. Mostly they include limited text on occupational safety issues, and focus on one specific group of workers; for example, the National Youth Policy 2015 contains guidance on the safety of young people in the workplace. It is stated under Subsection 10(b)15, “*Guaranteeing safe, healthy and decent work and promoting the programme of establishing the labour rights, emphasis shall be laid on promoting the youth labour*.”

The Employment Policy 2014 considered contribution to the national economy by facilitating decent and safe employment for the labour force (Subsection 7.1). However, it does not outline any policies on occupational safety except for the agriculture sector, which states, “*To ensure biological safety in the agriculture sector and physical safety of workers, use of pesticides will be minimized and use of safety measures during pesticide application will be made mandatory*” (Subsection 10.13).

Overall, most of the documents found by this review, across any of the injury locations, do not contain clear provisions or promote the collection and dissemination of data on injuries. Similarly, policy documents do not consider the creation of mechanisms for the coordination of policy implementation and support programmes with the authority to undertake periodic reviews.

### Legislative provisions on first response

We sought to identify legislation relevant to the provision of first aid services, emergency health care and availability of health services. This area also includes the management and availability of fire engines in the case of any fire events and disaster emergency such as earthquakes. We identified 18 legislative documents developed by seven ministries: Health and Population; Women, Children and Senior Citizens; Labour; Home Affairs; Physical Infrastructure and Transport; Education; and Energy. In these documents, first response is presented as first aid or emergency or primary care or by describing working with the ambulance service. We found that the reviewed documents mostly described the responsibilities of hospital facilities or ambulance services, and did not include local arrangements such as community volunteers and trained first aid or first response activities.

The Public Health Service Act 2018 defined emergency health services as “*the initial and immediate service to be provided as it is necessary to free the lives of the persons from risk, save the lives or organs from being lost, whose lives are in the risky condition upon falling into unexpected incident or emergency condition*” (Chapter 1, Section 2.a). This Act has criminalized the denial of health service and emergency care to visitors. Section 52 (Offence) in Chapter 8 stipulated “*to make refusal by a health institution to provide basic health service and emergency treatment available at the health institution shall be deemed as a committed offence by such a person refusing the service*.” The Ambulance Service Operation Guideline 2016 classified ambulances into three categories: A (advanced life support), B (basic life support) and C (common life support). This Guideline clearly stated that ambulance service should be provided free of cost to those who are too poor to pay. With regard to qualifications, Section 11.1(d) of this Guideline stipulates that “*ambulance drivers should be trained in first aid course prescribed by national emergency treatment coordination committee*.” Therefore, with the exception of these two documents which provide grounds for the development of actual prehospital care systems, all others have provisions for making necessary arrangements for providing first aid or prehospital care to the injured person. For example, the Labour Rules 2018 provisioned in Rule 49.1, “*(first aid) the employer shall make arrangements for immediate first aid available within the premises of the Business*.”

### Policies related to first response

We found 13 policies produced by various agencies that included provisions for first response to injuries. Policies such as the National Health Strategy, National Health Policy, National Development Plan and the Disaster Risk Reduction Policy focused on two main issues, the first being the development of the first response system with the establishment of emergency services at local, provincial and city levels. The second issue addressed was improving access to prehospital care for injured persons, which is also mentioned in the Public Transport Code of Conduct. These are illustrated in the following excerpts from the current National Health Policy (2019) Section 6: “*Targeting road crashes occurring across major highways, trauma centres will be established to provide immediate treatment to the injured*” (Section 6.3.2). “*At least one Ambulance of specified standards, classification and equipped with modern technology will be arranged to each local/Palika level*” (Section 6.3.3). “*To provide emergency health services and rescue from remote places Air Ambulance will be developed*” (Section 6.3.4). “*To establish emergency health care fund and operationalize after formulation of Emergency Health Care Fund procedures*” (Section 6.3.5). “*Compulsory life support training will be provided to physicians, nurses and other health workers to make world-class emergency care quality services*” (Section 6.3.6).

The current National Development Plan (15th Plan, 2019–2024) has addressed some essential aspects of the first response system by allocating financial resources to establish basic emergency surgery and primary trauma care centres at grassroots levels. It aims to “*establish basic emergency surgery and primary trauma care centre in every ward*” (Chapter 7.3 Health & Nutrition, Strategy 4, Action 4.1). It also has a target of “*integrating ambulance service and trained health professional mobilization system in coordination among federal, provincial and local government during disaster*” (Strategy 8, Action 8.6). The 15th National Plan has also envisioned sports clinics to deal with sports injuries.

These objective plans are the outcomes of national health policy, strategy and disaster risk reduction policy. The policy documents included in this review did not include provisions for the creation of a universal telephone number for emergencies, the availability of immediate response teams during emergencies, or the availability of fire engines and trained fire brigades.

### Comparison with WHO recommendations

The provisions contained within the 62 included policy documents were compared against the evidence-based recommendations for injury prevention and first response published by WHO.

**Fig. 4:**
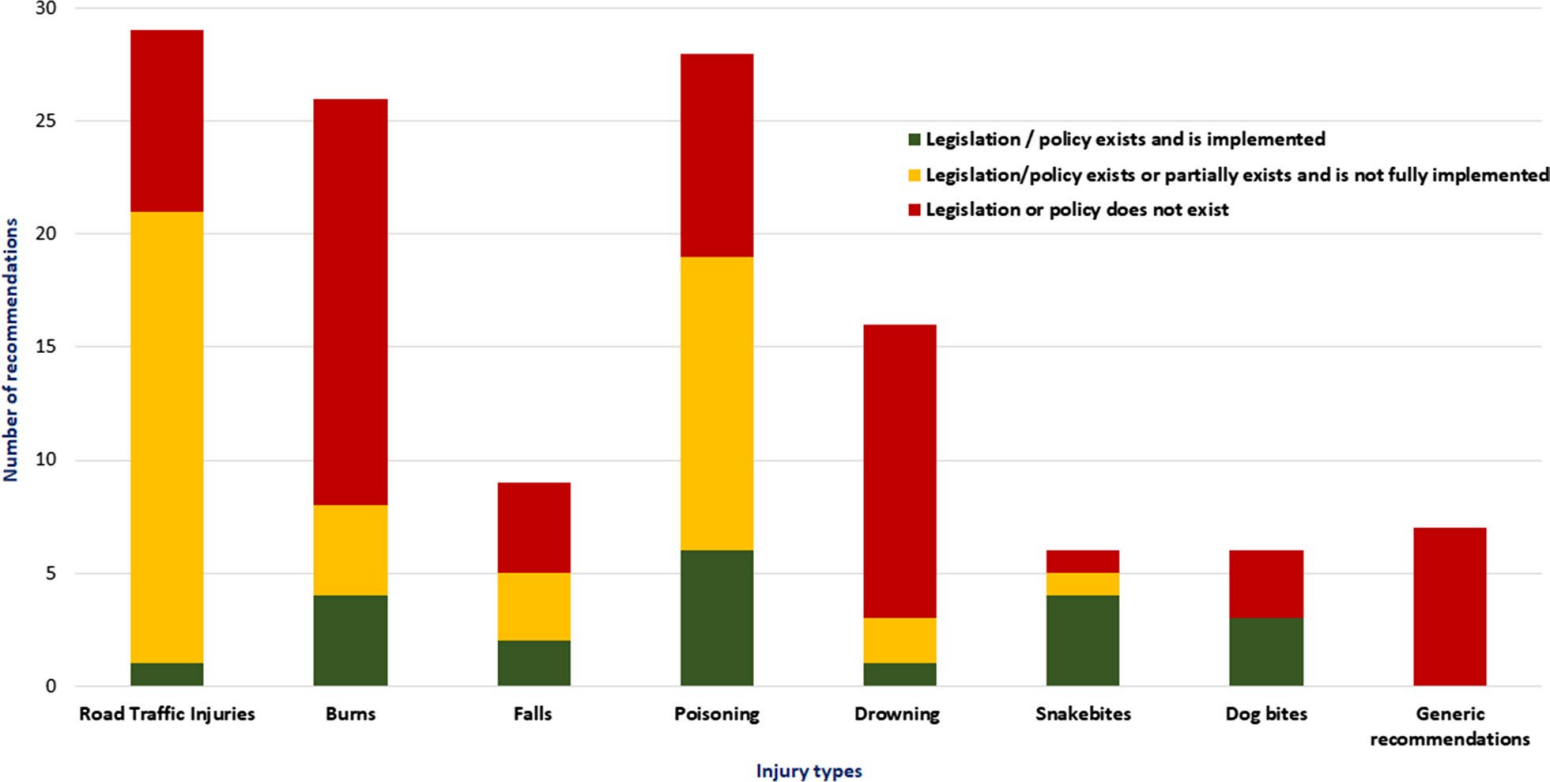
Status of laws and policies compared to WHO recommendations for injury prevention and control

Of 127 strategic recommendations by WHO that provided an area for policy or legislative focus, 21 (16.5%) were considered adequately met by Nepali policy and legislation, 43 (33.9%) were considered partially met and 63 (49.6%) were not met (Fig. 4 and Table 1).

**Table 1:**
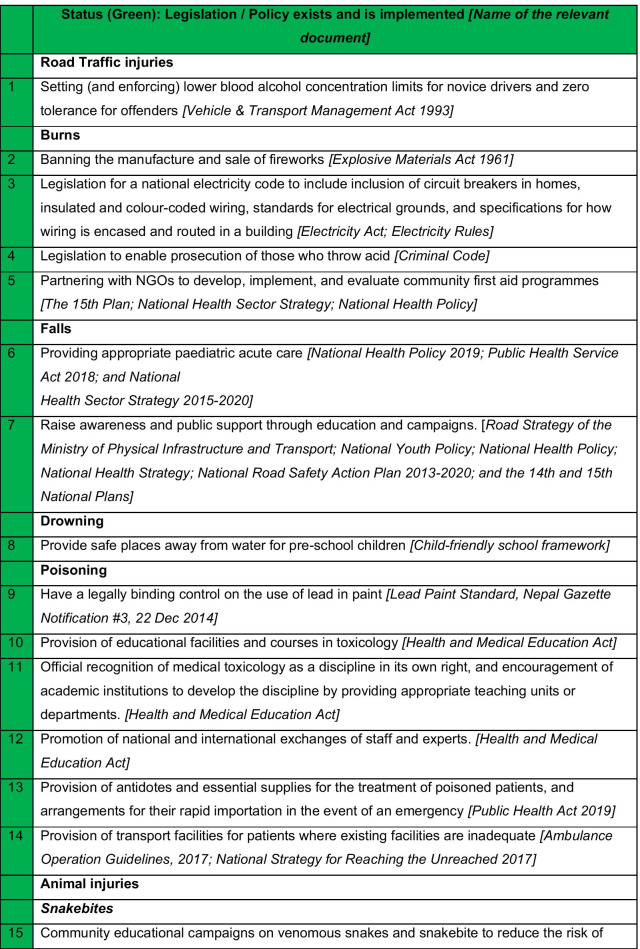

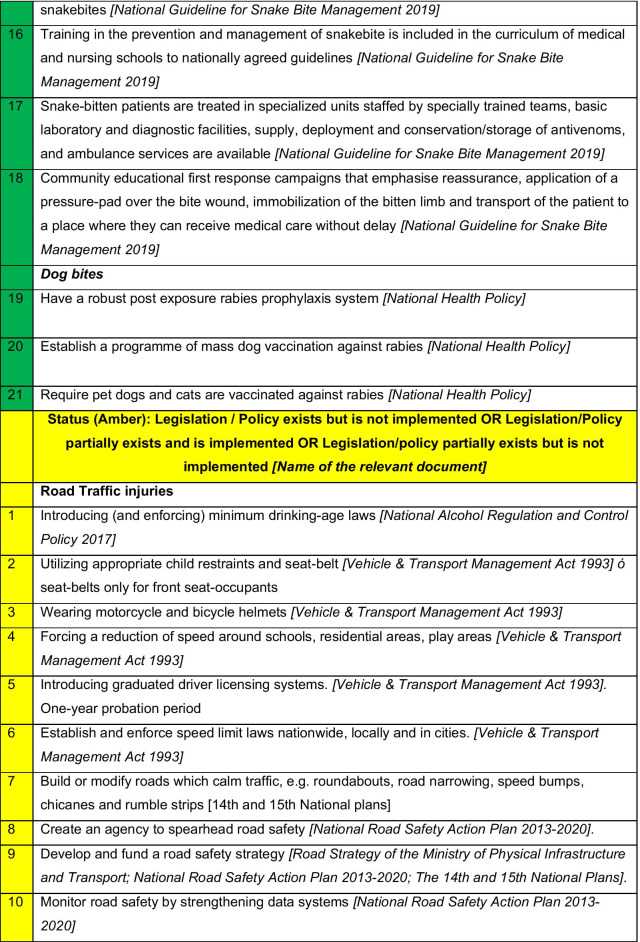

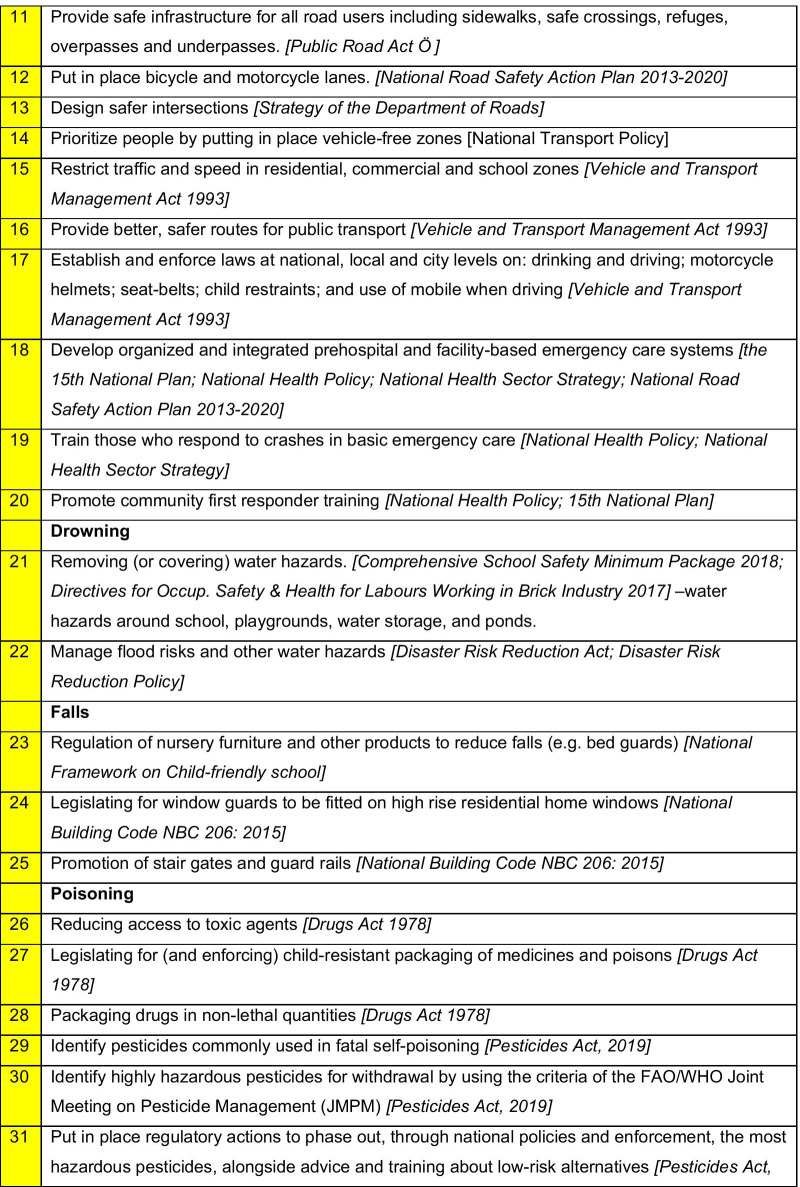

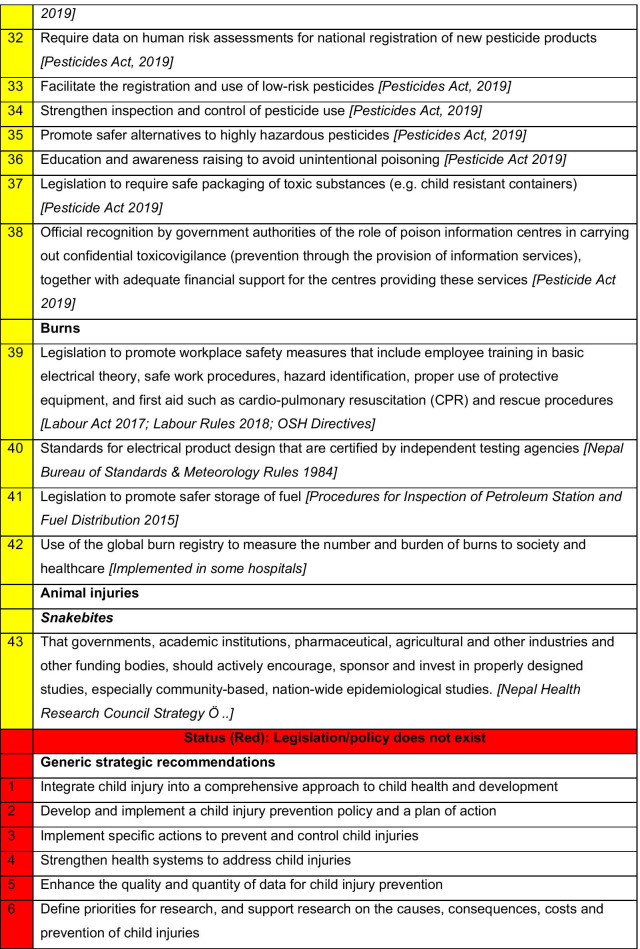

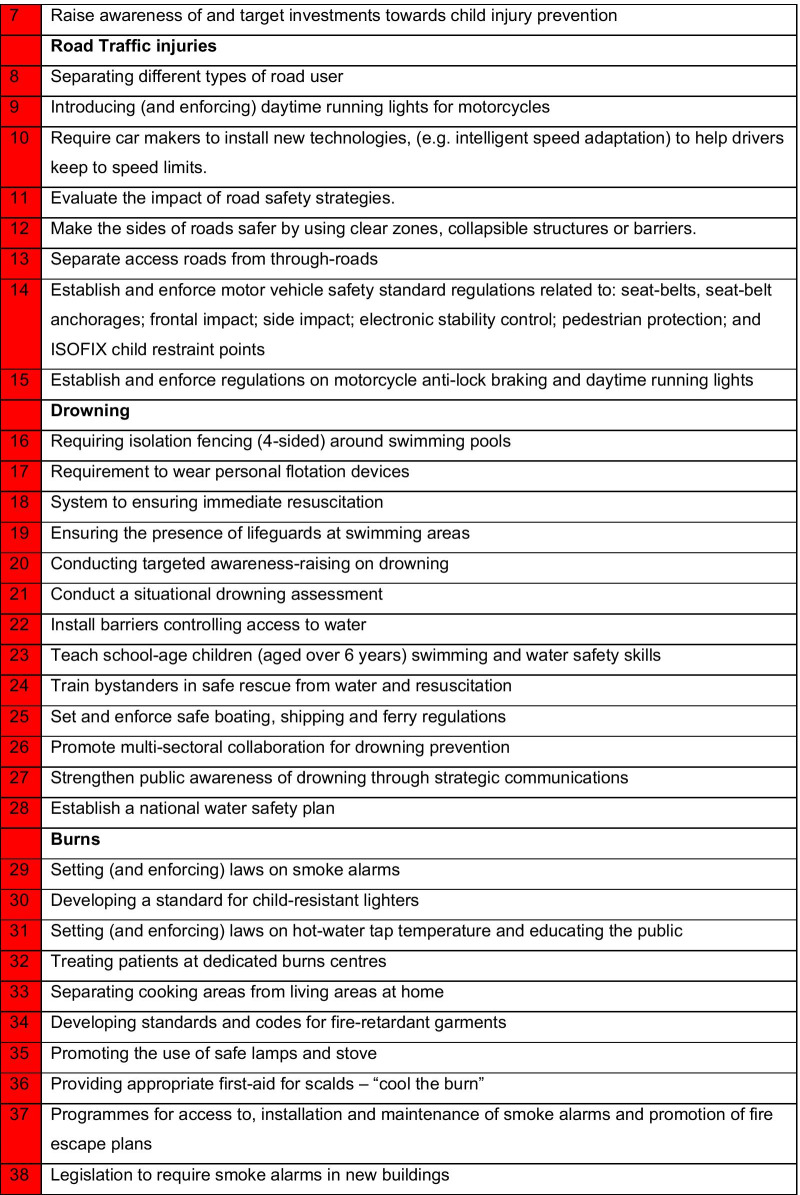

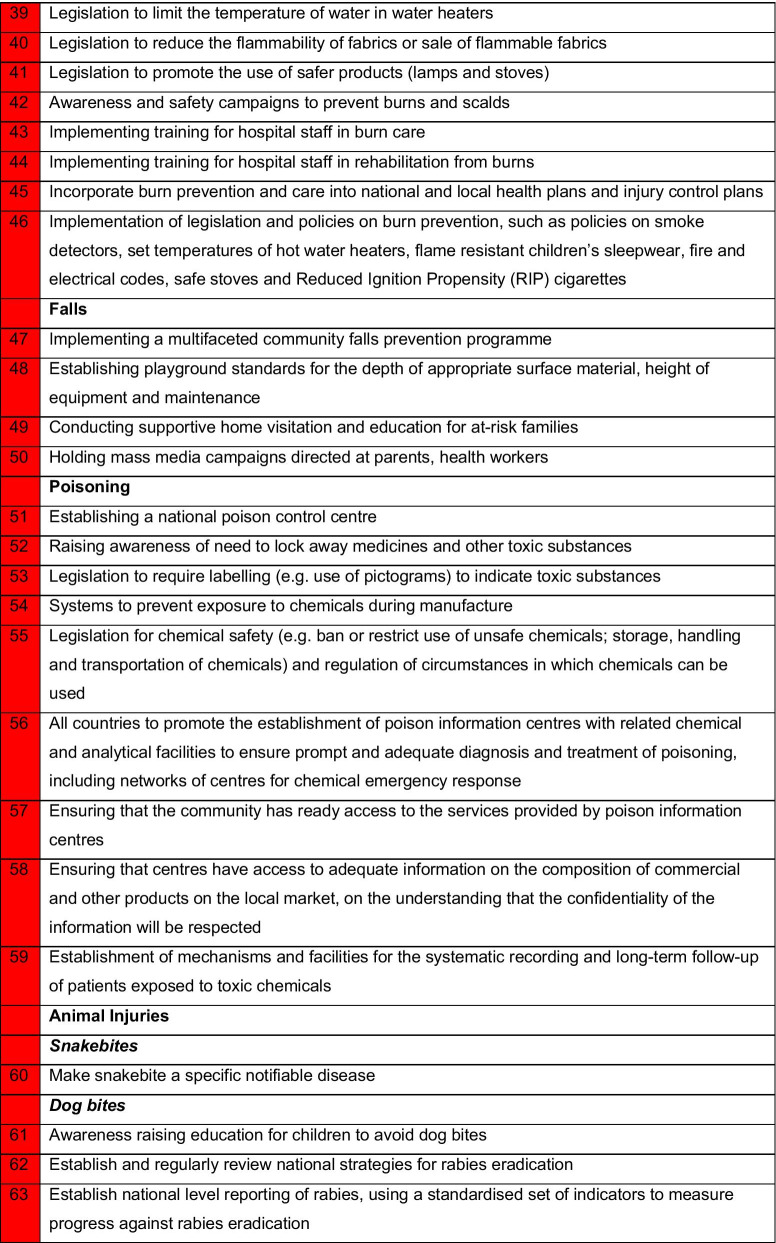
Comparison of WHO recommendations with existing policies in Nepal

Road Traffic Injuries: World report on road traffic injury prevention [31]; ‘Effective’ and ‘Promising’ strategies recommended in the World Report on Child Injury Prevention 2008 [32]; WHO Guidelines for poison control [52]. Save LIVES: Road Safety Technical Package 2017[55]. Burns: Plan for Burn Prevention and Care [53]; WHO Burn prevention and lessons learned [57]; Global Burn registry [58]. Drowning: WHO Preventing drowning: an implementation guide [56]. Poisoning: WHO Preventing Suicide; a resource for pesticide registrars and regulators [59]; WHO Regulations and control on lead paint; WHO/UN Environmental programme/International Labour Organisation. Guidelines on the prevention of toxic exposures [60]; Animal injuries: WHO Guidelines for the management of snake-bites in south-east Asia [54]; Prevention of dog bites and rabies [61]; FAQs on Rabies; Driving progress towards rabies eradication: second workshop on rabies management [62]

## Discussion

It is a fact that injury is a neglected but major global health problem [3]. Despite the evidence that injury prevention legislation and enforcement are highly efficacious and cost-effective interventions [43], injury prevention is often neglected and underfunded as a public health issue [3]. That said, most countries of the world have some laws and regulations that address violence and injury [23, 24, 44]. This study identified 62 national laws and policy documents relevant to various injuries and first response.

The Constitution of Nepal 2015 stipulates health as a fundamental right. Although not solely responsible for injury prevention and safety promotion, a dozen ministries have made legal provisions relevant to injury prevention. As elsewhere in the world, the majority of such documents were related to the prevention of road traffic injuries [38], driven by the United Nations Decade of Action for Road Safety (2011–2020) and WHO’s *World report on road traffic injury prevention* [31]. We found fewer policy documents on home, school and occupational injuries. Although Nepal lacks a robust first response system to respond to injury at the scene of the incident and care for injured persons, there exist some provisions that support first-aid and emergency medical services [45]. Altogether 35 (out of 62 included) documents were formulated (revised or updated) on or after 2015 (Fig. 2). The majority of the included legislative and policy documents were initiated during the unitary structure of State, that is, prior to 2015. It is therefore not surprising that almost all of the documents have mandated responsibilities to the federal (central) government. The year 2015 marked two major events: major earthquake and promulgation of the Constitution of Nepal.

The Constitution of Nepal 2015 has resulted in a new federal structure. Three layers of government can formulate their own laws on issues stated in an exclusive list of rights. For other issues, the federal government formulates laws with national standards, the provincial government formulates laws with contextual details in line with the federal act, and local government adopts laws and procedures to deliver the services as provisioned in federal and provincial laws. Reducing the burden and risk of injuries at home, at school, on the road and in the workplace not only requires the formulation of acts, policies and strategies but also requires a planned and financed approach to their implementation. In view of the new federalized system of government, it is now necessary to review those laws and policies that do not identify responsible agencies and actions at the provincial and local government level. Mechanisms are also needed for coordinating action across government levels and between multiple agencies and stakeholders. Lack of coordination is a major impediment in successful implementation of plans or laws in societies in different parts of the world [46, 47].

All types of injuries are associated with particular risk factors, and some population groups are more at risk of certain injuries than others. For example, road traffic injuries are more common in young adults, while injuries occurring at home are more common in preschool children and older people. We found that the injury-related laws and policies in Nepal were largely focused on changes to infrastructure, through design and construction, rather than on influencing public behaviour or vulnerable groups, the exception being those that prevent occupational injuries. Acts, laws and policies adopted since 2015 in compliance with the new constitution and federal structure involve a broader range of agencies, address injuries occurring in a wider range of locations (including workplaces and schools) and consider a wider range of vulnerable groups. Despite this, home and school injuries were less well addressed than injuries occurring on the road or at work.

In existing legislation and policies, injuries tend to be considered as an outcome of unexpected “accidents” rather than as a predictable outcome arising from absence or inadequacy of preventive measures. This lack of a focus on anticipatory action is also exemplified in the limited laws and policies relating to the provision of first response services. Nepal does not have an effective emergency medical service, there is no national emergency phone number, and ambulance services are largely private sector-operated systems to convey patients to hospital, rather than provide prehospital care that can save lives and limit disability following an injury. Countries with the lowest injury rates in the world, such as Sweden or the Netherlands, are ones that adopt a “safe systems” approach to injury [48]. A safe system is one where multiple agencies and sectors work collaboratively to contribute to an environment that is forgiving of human error and behaviours that place individuals at risk of harm [48].

In this review we found that multiple institutions/agencies in Nepal were identified as responsible for the formulation and implementation of laws and policies on injury prevention and first response. According to WHO’s framework for violence and injury prevention [23, 24], a country’s health ministry should have a mandate for injury prevention, but it is not essential that it take the lead. Some areas of injury prevention may primarily involve other sectors, such as the ministry of transport (for road injuries) or the ministry of labour (for occupational injuries) [23]. In Nepal, whilst ministries may be named as lead agencies, we found a lack of evidence that such responsibilities were followed through, with no designated individual to lead, or no budget on which to implement and ensure enforcement of the content of the legislation or policy. Several factors including lack of leadership and insufficient or nonexistent budget are identified as major challenges for the implementation of health policies [49]. Effective injury prevention requires public education to raise awareness of injury risks and ways that people can keep themselves and others safe from harm. However, education alone does not change behaviour at a population level, which is more likely to be achieved through environmental change and enforced legislation [50]. The ministry of health, being the focal agency for injury prevention, should also establish an effective first response system for injuries as a primary responsibility [23]. However, we found no designated leadership for this area in Nepal in our review. On the basis of the findings of this study, short- and medium-term actions are recommended.

Federal, provincial and local governments should work together to agree how existing legislation and policies supporting injury prevention, and first response to injuries, should be implemented, enforced and monitored through the federalized system of government. Federal agencies must develop partnerships and mobilize non-state actors and the private sector to enable the implementation of existing policies at the provincial and local levels [short-term action].

Federal government:

- Existing legislation and policy for injury prevention and first response should be updated where it is found to be incomplete or out of date, or to reflect evidence of effective interventions where available [short-term action].
- Federal government should deliver awareness campaigns to inform the public regarding any changes to existing legislation and policy [short-term action].
- Federal government should develop, implement and use data systems that enable the collation of high-quality information on injuries to inform decision-making [short-term action].
- New laws and policies should be considered that orchestrate the entire system, from designating a lead agency, identifying the causes of injuries, to financing evidence-based implementation mechanisms, with the goal of preventing injuries in general or injuries that occur on the road or at home, school or work, including the arrangements for first response or prehospital care systems [medium-term action].
- Federal government should develop and depute human resources for the implementation and enforcement of existing injury prevention and first response activities [short-term action] and consider it in any new legislation and policy that is developed in the future [medium-term action].
  Provincial governments:
- Provincial governments should formulate laws and policies suitable to their needs that enable them to implement and monitor national policies and strategies to prevent injuries on the road, or at home, school or work, and to ensure that an adequate first response to injuries is available [short-term action].
  Local governments:
- Local governments should ensure that existing legislation and policies are implemented at a local level through the engagement of local stakeholders [short-term action].
- Local governments should formulate legislation and policies, as authorized by the Local Government Operation Act, to enable them to implement and monitor national and provincial strategies for injury prevention and first response [medium-term action].

### Strengths and limitations of the study

To our knowledge, this is the first critical review of policies supporting injury prevention and first response in Nepal. We included the broad definition of injuries as described by WHO. We involved stakeholders at the outset of the process, confirming with them the criteria for including documents in the review, and enabling us to identify the agencies responsible for different legislation or policies related to injury prevention and first response. Ascertainment of the final list of included documents, data extraction and writing of the report were completed in collaboration with an academic expert in law in Nepal. Our data extraction framework was piloted before being applied consistently across all included documents. We used WHO’s standard recommendations as best practice to compare against the provisions in the included documents from Nepal.

Although we used a range of methods and sources to identify documents for consideration in this review, it is possible that we might have missed some documents, for two reasons: First, in the absence of a national repository of legal documents, they were identified from multiple agencies (ministries, departments, commissions or websites) [51]. Secondly, we are aware of different perceptions among stakeholders regarding what constitutes “an injury”. We are aware that in Nepali society, injuries that do not leave a physical mark on the body (such as poisoning or drowning) may not be considered injuries. Consequently, some stakeholders may not have suggested documents that might have met our inclusion criteria. This review does not include intentional or violent injuries. We had originally planned to conduct a second meeting of stakeholders to discuss our proposed recommendations, but this was not possible due to government restrictions on meetings during the 2020 COVID-19 pandemic.

## Conclusions

This review showed that a wide array of agencies in Nepal are mandated to work on injury prevention and first response; however, no lead agency exists for these functions. Consequently, there is no clear leadership for the monitoring of injury burden or for the development of national injury prevention policy and strategy. A mechanism that facilitates coordination among different stakeholders is equally needed to accelerate such endeavours.

The documents included in this review mostly failed to identify the main causes of the injuries or the factors that make some individuals more vulnerable to particular types of injuries. WHO has published information on interventions proven to reduce the incidence and severity of injuries [18, 31, 32, 52–56]. There are some areas where Nepal has made good progress with the implementation of evidence-based prevention interventions. However, there is ample opportunity to address areas where no action has been taken, or to strengthen action where actions have only partially been formulated or implemented.

Legislation and policy must consider the financial implications, and promulgation should occur with adequate funding to employ staff to implement and enforce laws. Financial investment is also required for the infrastructure and human resources to support the monitoring of injury prevention efforts. Globally, the economic case for investment in prevention of injuries is advancing, and within Nepal, the evidence base illustrating the burden of injuries on Nepali society is growing.

## Supplementary information

**Additional file 1:** Supplementary table 1. Lists of included and excluded documents.

**Additional file 2:** Relevant provisions in the Constitution and the Local Government Operation Act.

## Data Availability

None.

## Abbreviations

A-CHIPP: Assessment of child injury prevention policies
ICESCR: International Covenant on Economic, Social and Cultural Rights
MDG: Millennium Development Goals
MTVM: Motor vehicles and transport management
RAIMS: Road accident information management system
SSDP: School Sector Development Plan
