# Supplementary material for: The prevention of – and first response to – injuries in Nepal: a review of policies and legislation
Source: Health Res Policy Syst. 2021 Apr 14;19:65. doi: 10.1186/s12961-021-00686-1 (PMC8045995; doi:10.1186/s12961-021-00686-1)
Supplement: Supplementary file 1 — Additional file 1: Supplementary table 1. Lists of included and excluded documents. [file 12961_2021_686_MOESM1_ESM.docx]

## Supplementary table 1. Lists of included and excluded documents

## A. Detailed list of included documents

| **Name, Year (AD)** | **Originating agency** | **Enforcing agency** | **Injury in scope** | **Risk factors** | **Preventive measures** |
| --- | --- | --- | --- | --- | --- |
| **The Constitution of Nepal, 2072 (2015)** | **GoN** |  | **all** |  |  |
| **Local Government Operation Act, 2074 (2017)** | **MoFAGA** |  | **all** |  |  |
| Act Relating to Children 2075 (2018) | MoWCSC | MoWCSC, NCRC | Home, School & Road Injuries | Policy Enforcement | Adoption of Policies |
| Ambulance Service Operation Guideline, 2073, (2016) | MoHP | DoHS | Road injuries; disasters |  |  |
| Bus Body Building Directives, 2074 (2018) | DoTM | DoTM | Road Injuries | Structural Risk | Improving Structures |
| Child Labour (Prohibition & Regulation) Regulation 2061 (2005) | MoLESS | Labour Office | Occupational Injuries | Machinery Risk & Less Safety Equipment | Punitive Actions & Safety Equipment |
| Child Labour Prohibition & Regulation Act 2056 (2000) | MoLESS | Labour Office | Occupational Injuries | Machinery Risk & Less Safety Equipment | Punitive Actions & Safety Equipment |
| Comprehensive School Safety Implementation Guidelines, 2075 (2018) | MoEST | DoE | School Based Injuries | Structural Risk & Less Safety Equipment | Adoption of Policies & Safety Equipment |
| Directives on Regulating Weigh Load of Transporting Vehicle, 2074 (2018) | DoTM | DoTM | Road Injuries | Structural Risk | Punitive Actions |
| Driving Training Centre Operation Directives, 2074 (2018) | DoTM | DoTM | Road Injuries | Behaviours Risk | Improving Structures |
| Driving Training Curriculum, 2074 (2018) | DoTM | DoTM | Road Injuries | Machinery Risk & Behaviour Risk | Awareness |
| Drugs Act 2035 (1979) | MoHP | DoD | Poison | Structural Risk & Behaviour Risk | Punitive Actions & Safety Equipment |
| Electricity Act, 2049 (1992) | MoEWRI | NEA | Home Injuries | Structural Risk | Improving Structures |
| Electricity Rules, 2050 (1993); amendment 2066 (2010) | MoEWRI | NEA & Power Plan Owners | Home Injuries & Occupational Injuries | Structural Risk & Less Safety Equipment | Improving Structures & Safety Equipment |
| Explosive Materials Act 2018 (1961) | MoHA | DAO | Fire Injuries | Fire arms, explosive materials & fireworks | Regulation of Fireworks sell & distribution |
| Five-year Strategic Plan for Transport Development 2073-2078 (2016-2021) | MoPIT | MoPIT | Road Injuries | Structural Risk & Less Safety Equipment | Adoption of Policies |
| Foreign Employment Act 2063 (2007) | MoLESS | DoFE | Occupational Injuries | Structural Risk, Machinery Risk & Behaviour Risk | Punitive Actions |
| Health Care Technology Policy, 2062 (2006) | MoHP | MoHP | Occupational Injuries | Machinery Risk | Adoption of Policies & Safety Equipment |
| Health Insurance Act 2074 (2017) | MoHP | Health Insurance Board | General |  |  |
| Labour Act, 2075 (2018) | MoLESS | DoL | Occupational Injuries | Structural Risk, Machinery Risk & Less Safety Equipment | Adoption of Policies, Punitive Actions & Safety Equipment |
| Labour Policy 2063 (2006) | MoLESS | MoLESS | Occupational Injuries | Machinery Risk & Less Safety Equipment | Improving Structures & Safety Equipment |
| Labour Rules, 2075 (2018) | MoLESS | DoL | Occupational Injuries | Structural Risk, Machinery Risk & Behaviour Risk | Adoption of Policies, Punitive Actions & Safety Equipment |
| Master Plan for Strategic Road Network, (2062) 2005 | DoR | DoR | Road Injuries | Structural Risk | Improving Structures |
| Medium & Light Motor Vehicle Inspection Manual, 2074 (2018) | DoTM | DoTM | Road Injuries | Machinery Risk | Improving Structures |
| Medium & Small Vehicle Testing Manual, 2074 (2018) | DoTM | DoTM | Road Injuries | Machinery Risk | Improving Structures |
| Model School Development & Operation Guideline, 2073 (2017) | MoEST | DoE | School Based Injuries | Structural Risk | Adoption of Policies & Safety Equipment |
| Motor Vehicle & Transport Management Rules, 2054 (1997); amendment 2066 (2010) | MoPIT | DoR & Traffic | Road Injuries | All | All |
| Motor Vehicles & Transport Management Act, 2049 (1993); amendment 2050 (1993) | MoPIT | DoR & Traffic | Road Injuries | All | All |
| National Adolescent Health & Development Strategy 2074 (2018) | MoHP | MoHP | Road Injuries, Occupational Injuries & School Injuries | Structural Risk & Behaviour Risk | Improving Structures & Awareness |
| National Children Policy 2069 (2013) | MoWCSC | MoWCSC, NCRC | School Based Injuries & Home-Based Injuries | Policy Enforcement | Adoption of Policies |
| National Criminal Code 2074 (2017) | MoLJPA | Police & Court | Fire, Animal Attack, Acid Attack | Fire Arms, Poison, Acid Burn | Punitive Actions |
| National Employment Policy 2071, (2014) | MoLESS | MoLESS | Occupational Injuries | Machinery Risk & Less Safety Equipment | Safety Equipment |
| National Framework of Child-Friendly School for Quality Education, 2067 (2010) | MoEST | DoE | School Based Injuries | Structural Risk | Adoption of Policies & Safety Equipment |
| National Guidelines for Snakebite Management in Nepal 2019 | MoHP | DoHS | Snake Bite | Snake Bite Response | Protocol Application |
| National Health Policy 2071 (2015) | MoHP | MoHP | Road Injuries, Occupational Injuries & School Injuries | Structural Risk, Machinery Risk, Behaviour Risk, Policy Enforcement | Adoption of Policies |
| National Health Policy, 2076 (2019) | MoHP | MoHP | Road Injuries & School Based Injuries | Behaviours Risk & Policy Enforcement | Adoption of Policies & Safety Equipment |
| National Policy for Disaster Risk Reduction, 2075 (2018) | MoHA | MoHA | Road Injuries & Home Injuries | Policy Enforcement | Adoption of Policies & Safety Equipment |
| National Road Safety Action Plan (2070-2077), 2070 (2013-2020), 2013 | MoPIT | MoPIT | Road Injuries | ALL | All |
| National Transport Policy, 2058 (2001) | MoPIT | MoPIT | Road Injuries | Structural Risk, Machinery Risk & Behaviour Risk | Improving Structures, Punitive Actions & Awareness |
| National Urban Development Strategy 2074 (2017) | MoUD | DUDBC | Home Injuries | Policy Enforcement | Adoption of Policies |
| National Youth Policy, 2072 (2015) | MoYS | NYC | Occupational Injuries | Machinery Risk & Behaviour Risk | Safety Equipment, Behaviour Change |
| Nepal Health Sector Strategy, (2072-2077), 2072 (2015-2020), 2015 | MoHP | MoHP | Road Injuries | Policy Enforcement | Adoption of Policies |
| Nepal Road Standard, 2070 (2013) | DoR | DoR | Road Injuries | Structural Risk & Behaviour Risk | Improving Structures & Safety Equipment |
| Occupational Safety & Health for Labours Working in Brick Industry, 2074 (2017) | MoLESS | DoL | Occupational Injuries | Structural Risk, Machinery Risk & Less Safety Equipment | Adoption of Policies & Safety Equipment |
| Pesticides Management Act 2076 (2019) | MoALD | Centre for Plants Quarantine & Pesticide Management | Poison | Poison | Labelling, Storage, Registration, Inspection, Control |
| Procedures for Inspection & Maintenance of Bridges (2003) | DoR | DoR | Occupational Injuries | Structural Risk & Behaviour Risk | Adoption of Policies & Safety Equipment |
| Procedures for Removing Obstruction in Highway 2074 (2018) | MoHA | DAO | Road Injuries |  |  |
| Public Health Service Act, 2075 (2018) | MoHP | Local, province & federal govts. | General |  |  |
| Public Road Act, 2031 (1974) | MoPIT | DoR | Road Injuries | Structural Risk & Behaviour Risk | Punitive Actions |
| Public Transport Code of Conduct, 2068 (2012) | DoTM | Monitoring Committee | Road Injuries | Behaviours Risk | Awareness |
| Road Board Act 2058 (2001) | MoPIT | Road Board | Road Injuries | Structural Risk | Adoption of Policies |
| Road Board Directives 2061 (2004) | Road Board | Road Board | Road Injuries | Structural Risk | Improving Structures |
| Route Determining Directives, 2074 (2018) | DoTM | DoTM | Road Injuries | Structural Risk | Adoption of Policies |
| School as Zone of Peace National Framework & Implementation Guideline, 2068 (2011) | MoEST | SMC/CPC | School Based Injuries | Behaviours Risk | Policy Enforcement |
| School Bus Directives, 2074 (2018) | DoTM | DoTM | Road Injuries | Structural Risk | Improving Structures & Safety Equipment |
| School Sector Development Plan (SSDP) 2073/74-2079/80 (2016/17-2022/23) | MoEST | DoE | School Based Injuries | Structural Risk | Adoption of Policies |
| The Building Act, 2055 (1998); amendment 2066 (2010) | MoPIT | Local Government | Home & School Injuries | Structural Risk | Improving Structures |
| The Department of Roads Strategy, 2052 (1995) | DoR | DoR | Road Injuries | Structural Risk & Machinery Risk | Improving Structures, Adoption Policies |
| Transport Management Procedural Directives, 2060 (2003); amendment 2074 (2018) | DoTM | DoTM | Road Injuries | Enforcement | Adoption of Policies |
| Workshop & Garages Operation Directives, 2074 (2018) | DoTM | DoTM | Road Injuries | Machinery Risk | Improving Structures |
| 14^th^ Periodic Plan, 2073/74-2075/76 (2016/17-2018/19) | NPC | MoPIT & MoLESS | Road Injuries & Occupational Injuries | Structural Risk, Machinery Risk & Safety Equipment | Adoption of Policies & Safety Equipment |
| 15^th^ Five-year Plan, 2076-2081 (2019-2024) | NPC | MoPIT & MoLESS | Road Injuries, Home Injurie, Occupational Injuries, School Based Injuries | Structural Risk, Machinery Risk, Behaviour Risk, Policy Enforcement | Improving Structures, Adoption Policies, Punitive Actions |

DAO= District Administration Office DoE= Department of Education

DoFE= Department of Foreign Employment DoHS= Department of Health Services

DoL= Department of Labour DoR= Department of Roads

DoTM= Department of Transport Management DUDBC= Department of Urban Development & Building Construction

GoN = Government of Nepal MoEST= Ministry of Education, Science & Technology

MoEWRI= Ministry of Energy, Water Resource & Irrigation MoFAGA = Ministry of Federal Affairs and General Administration

MoHA= Ministry of Home Affairs MoHP= Ministry of Health & Population

MoLESS= Ministry of Labour, Employment & Social Security MoLJPA= Ministry of Law, Justice & Parliamentary Affairs

MoPIT= Ministry of Physical Infrastructure & Transport MoWCSC= Ministry of Women, Children & Senior Citizen

NCRC = National Child Rights Council NEA= Nepal Electricity Authority

NPC= National Planning Commission NYC= National Youth Council

SMC = School Management Committee

## B. Detailed list of excluded documents

| **Agency** | **Document downloaded** | **Injury Provisions (Yes/No)** |
| --- | --- | --- |
| Ministry of Physical Infrastructure & Transport | Good Governance (Management and Operation) Act, 2064 | No |
|  | Environment Friendly Motor Vehicle and Transport Policy, 2071 | No |
| Department of Transport Management | Directives for management of Practical Exam for distribution of motor vehicle license, 2069 | No |
|  | [Standard for Transfer and Posting, 2071](https://dotm.gov.np/uploads/files/saruwa_standard_2071.pdf) | No |
|  | Workshop Standard, 2071 | Replaced by new |
| Department of Road | Public Procurement Act, 2007 AD | No |
|  | Public Procurement Regulations, 2007 AD | No |
|  | Manual of Standard Tests, 2016 | No |
|  | Delineation Measures, 1996 | No |
|  | Design safe side drains, 1996 | No |
| Ministry of Health and Population | National Adolescent Health and Development Strategy, 2057 (2000) | Replaced by new |
|  | Health Sector Strategy: An Agenda for Reform (2004) | No |
|  | Health Sector Gender Equality and Social Inclusion Strategy, 2066 (2009) | No |
|  | Health Sector Information System National Strategy, 2063 | No |
|  | National Blood Policy, 2050 (1993) | No |
|  | National Health Research Policy, 2003 | No |
|  | National Blood Transfusion Policy, 2071 (2014) | No |
|  | National Strategy for Reaching the Unreached, 2073-2088 (2016-2030) | No |
|  | Policy, Strategy and 10 Year Action Plan on Disability Management, 2073-2082 (2016-2025) | No |
|  | Multisectoral Action Plan for the Prevention & Control of Non-Communicable Diseases (2014-2020) | No |
|  | Nepal Health Sector Strategy- Implementation Plan (2073-2078), 2073 (2016-2021), 2016 | No |
|  | Early Warning and Reporting System Guidelines, 2016 | No |
|  | Nepal Health Service Act, 2053 | No |
| Ministry of Labour, Employment and Social Security | Act Relating to Employment Rights, 2018 | No |
|  | Rules relating to Employment Rights, 2075 | No |
|  | Trade Union Act, 2049 | No |
|  | Trade Union Regulations | No |
|  | Labour Audit Standard, 2075 | No |
|  | OSH Standard for Noise and Light at Workplace, 2073 (2016) | No |
|  | National Master Plan on Elimination of Child Labour (2075-2085) | No |
|  | Social Security Scheme Procedural Guidelines 2075 (2018) | No |
| Ministry of Women, Children and Senior Citizens | National Policy Relating to Children, 2069 | No |
|  | Domestic Violence (Offence and Punishment) Act, 2066 | No |
|  | Workplace sexual harassment (prohibition) Act, 2071 | No |
|  | National Center for Educational Development, 2062 (2005) | No |
|  | National Policy and Plan of Action on Disability, 2063 (2006) | No |
|  | The Act Relating to Rights of Persons with Disabilities, 2074 (2017) | No |
|  | Special Education Policy, 2053 | No |
| Ministry of Urban Development | Guidelines on load bearing masonry, 2015 | No |
|  | Guidelines for Earthquake Resistant Building Construction: Low strength masonry, 2015 | No |
| Department of Urban Development and Building Construction | National Urban Policy, 2007 | No |
|  | Specifications of Building Construction (Civil) Works | No |
|  | Occupancy load, 1994 | No |
|  | Plain and reinforced concrete, 1994 | No |
|  | Sanitary and plumbing design requirement, 2003 | No |
|  | Seismic design of buildings in Nepal, 1994 | No |
| National Planning Commission | Civil Rights Act, 2049 (1993) | No |
|  | The Right to Employment Act, 2075 (2018) | No |
|  | Local self-governance rules, 2056 (1999) | No |
|  | Industrial Policy, 2011 | No |
|  | Special Education Policy, 2053 (1996) | No |
| Ministry of Education, Science & Technology | Comprehensive School Safety Communication & Dissemination Strategy, 2075 (2018) | No |
|  | Education Rules, 2059 (2002) | No |
|  | Policy Provision for Learning Without Fear, 2067 (2010) | No |
|  | Comprehensive School Safety Minimum Package 2075 (2018) | No |
| Ministry of Home Affairs | Disaster and Risk Management Act, 2074 (2016) | No |
| Ministry of Federal Affairs & General Administration | Fire Engine Operation and Management Directives, 2067 (2010) | No |

**(Total 57 documents)**

*The following documents however include the injury prevention provisions they do not provide any legal base for their enforcement. These are mainly good practice compilation and suggested recommended actions. Though it would be good to bring number of such standards as legally binding provisions, but currently they do not represent a legal status.*

| Architectural Design Requirements (National Building Code), 2072 (2015) |
| --- |
| Construction Safety, 2051 (1994) |
| Electrical Design Requirements for Public Buildings, 2060 (2003) |
| Fire Safety Codes 107 (Provisional Recommendations), 2051 (1994) |
| Health Management Information System Guidelines, 2073 (2015) |
| Identifying and Treating Accident, 2054 (1997) |
| Road Users Guide, 2056, (1999) |
| Safety Barrier, 2054 (1997) |

**(Total 8 Documents)**
