# Supplementary material for: The prevention of – and first response to – injuries in Nepal: a review of policies and legislation
Source: Health Res Policy Syst. 2021 Apr 14;19:65. doi: 10.1186/s12961-021-00686-1 (PMC8045995; doi:10.1186/s12961-021-00686-1)
Supplement: Supplementary file 2 — Additional file 2: Relevant provisions in the Constitution and the Local Government Operation Act. [file 12961_2021_686_MOESM2_ESM.docx]

**Additional file 2. Relevant provisions in the Constitution and the Local Government Operation Act.**

**Box 1: Provisions from The Constitution of Nepal that are related to Injury Prevention and First Response**

***Article 16. Right to live with dignity:*** *(1) Every person shall have the right to live with dignity.*

***Article 30. Right to Clean and Healthy Environment:*** *(2) The victim shall have the right to obtain compensation, in accordance with law, for any injury caused from environmental pollution or degradation.*

***Article 35. Right relating to health:*** *(1) Every citizen shall have the right to free basic health services from the State, and no one shall be deprived of emergency health services.*

***Article 39. Rights of the child:*** *(2) Every child shall have the right to education, health, maintenance, proper care, sports, entertainment and overall personality development from the families and the State.*

***Article 42: Right to Social Justice****: (5) The families of the martyrs who have sacrificed their life, persons who were forced to disappear, and those who became disabled and injured in all people's movements, armed conflicts and revolutions that have been carried out for progressive democratic changes in Nepal, democracy fighters, conflict victims and displaced ones, persons with disabilities, the injured and victims shall have the right to get a prioritized opportunity, with justice and due respect, in education, health, employment, housing and social security, in accordance with law.*

***Article 44. Rights of the consumer:*** *(2) A person who has suffered injury from any substandard goods or services shall have the right to obtain compensation in accordance with law.*

***Article 51 Policies of the State: (h): Policies relating to basic needs of the citizens:***

*(5) to keep on enhancing investment necessary in the public health sector by the State in order to make the citizens healthy,*

*(6) to ensure easy, convenient and equal access of all to quality health services,*

*(11) to manage unplanned settlement and develop planned and systematic settlement,*

*(14) to enhance investment in the transportation sector, while ensuring simple, easy and equal access of the citizens to transportation facilities, and to make the transportation sector safe, systematic and persons with disabilities friendly by encouraging public transportation and regulating private transportation, while according priority to the environment friendly technologies,*

*(15) to arrange for access to medical treatment while ensuring citizen's health insurance.*

***Article 51. Policies of the State: (i) Policies relating to labour and employment:***

*(2) to guarantee social security, while ensuring the basic rights of all labours, in consonance with the concept of decent labour,*

*(3) to abolish all forms of labour exploitation including child labour,*

*(5) to regulate and manage the sector in order to make foreign employment free from exploitation, safe and systematic and to guarantee employment and rights of the labours,*

***Article 51. Policies of the State: (j) Policies relating to social justice and inclusion:***

*(2) to make self-dependent the women who are vulnerable, subjected to social and family exclusion and victims of violence self-reliant by making their rehabilitation, protection and empowerment,*

| **Box 2: Provisions in the Local Government Operation Act that are related to Injury Prevention and First Response**  Section 3. Duties, responsibilities and authorities of Palika (rural and urban)    *Article #11. Authorities of Palikas*  1. Palikas (rural and urban) unitary authorities shall be as described in schedule 8 of the Constitution of Nepal.  2. Without contention to the principles of (1) above, the Duties, responsibilities and authorities of Palika (rural and urban) shall be as below:  a.2.h Disaster management related search, rescue, relief and rehabilitation  a.2.j Control stray animals  f.3 vital registration management for birth, death, marriage, divorce, migration and family records  f.5 Tasks related to local statistics and records collection  g.6 Approve construction of buildings according to national building codes and standards and monitoring.  g.7 Construction & maintenance and operation & management of Government building, schools, community buildings, seminar hall and other public buildings and structures  g.9 Develop policies, plans, programmes, activities and their implementation, regulation, monitoring and evaluation of safe settlements (housing).  i.8 Operation of blood transfusion service and local urban health services  k.1 Plan, implement, monitor and regulate the laws, policies, standards regarding local, rural and agriculture roads.  k.4 Management and regulation of Transport Safety  t. Disaster Management  t.1 Implementation, monitoring and regulation of the laws, policies, standards and plans regarding disaster management  t.2 Local level disaster preparedness and response planning, early warning system, search and rescue and relief aid storage, distribution and coordination.  t.7 Formulation, implementation, monitoring and regulation regarding local level disaster risk minimisation  t.10 Local level emergency work operation systems  t.11 Implementation of community-based disaster management programmes  4. Without contention to the principles in (3) the palikas (urban and rural) shall have the following duties, responsibilities and authorities under the jurisdiction of Federal and Provincial laws.  b.9 Local level Public Health Surveillance  b.15 Public health, emergency health and epidemic control planning and implementation  b.16 Disease control and prevention  b.17. Provide emergency health services and local health management  k. Permit to motor vehicles  k.1 Local level policies, laws, standards, plans, implementation and regulation related to transport management  k.3 Promote environmentally friendly, climate change adaptation, disaster risk response, disability- and gender-friendly local transport system  5. In addition to the authorities mentioned in (1) and (3) Palikas (Rural and Urban) shall have the following Duties, responsibilities and authorities:  c Transport services.  c.1 Develop policy, standards, plan, implementation, monitoring and regulation on medium capacity mass transit systems of local bus, trolley bus, and tram etc.  Article #12 Duties, responsibilities and authority of ward committees  2.c.1 Establish children's leisure parks  2.c.29 Make the ward as a child-friendly ward  2.d.6 Assist in control of electricity theft and stealing  Section 6 Article #26 Activities done in collaboration or partnerships  e. Fire engine and ambulance service operation |
| --- |
